# Polymers Containing Phenothiazine, Either as a Dopant or as Part of Their Structure, for Dye-Sensitized and Bulk Heterojunction Solar Cells

**DOI:** 10.3390/polym16162309

**Published:** 2024-08-15

**Authors:** Muhammad Faisal Amin, Amna Anwar, Paweł Gnida, Bożena Jarząbek

**Affiliations:** 1 Centre of Polymer and Carbon Materials, Polish Academy of Sciences, 34 M. Curie-Sklodowska Str., 41-819 Zabrze, Poland; 2 Joint Doctoral School, Silesian University of Technology, Akademicka 2a, 44-100 Gliwice, Poland; 3 Department of Chemistry, Quaid-i-Azam University, Islamabad 45320, Pakistan

**Keywords:** phenothiazine, polymers, dye-sensitized solar cells, bulk heterojunction solar cells, photovoltaic parameters

## Abstract

Potential photovoltaic technology includes the newly developed dye-sensitized solar cells (DSSCs) and bulk heterojunction (BHJ) solar cells. Owing to their diverse qualities, polymers can be employed in third-generation photovoltaic cells to specifically alter their device elements and frameworks. Polymers containing phenothiazine, either as a part of their structure or as a dopant, are easy and economical to synthesize, are soluble in common organic solvents, and have the potential to acquire desired electrochemical and photophysical properties by mere tuning of their chemical structures. Such polymers have therefore been used either as photosensitizers in dye-sensitized solar cells, where they have produced power conversion efficiency (PCE) values as high as 5.30%, or as donor or acceptor materials in bulk heterojunction solar cells. Furthermore, they have been employed to prepare liquid-free polymer electrolytes for dye-sensitized and bulk heterojunction solar cells, producing a PCE of 8.5% in the case of DSSCs. This paper reviews and analyzes almost all research works published to date on phenothiazine-based polymers and their uses in dye-sensitized and bulk heterojunction solar cells. The impacts of their structure and molecular weight and the amount when used as a dopant in other polymers on the absorption, photoluminescence, energy levels of frontier orbitals, and, finally, photovoltaic parameters are reviewed. The advantages of phenothiazine polymers for solar cells, the difficulties in their actual implementation and potential remedies are also evaluated.

## 1. Introduction

The development of green and cost-effective technology to produce clean energy using renewable resources is the ultimate target of today’s world. The increasing population of the world demands more energy now than in previous times, and therefore fossil fuel reserves are continuously depleting due to their lower availability and excessive use. This is an alarming situation, and alternate energy sources must be found to save the future of humanity. The sun, a natural fusion reactor, provides enough energy during one bright day to meet the energy demands of the current population for the next 27 years. It is a fact of immense interest that the energy provided by the sun in three days is equivalent to the energy stored in all fossil fuel reserves [1]. Therefore, researchers from all over the world are now focused on somehow capturing this freely available solar energy and utilizing it to meet current energy needs. Since the discovery of photovoltaic (PV) effects, continuous efforts have been made to utilize freely available solar energy by converting it into more usable forms [2]. With the advent of solar cell technology, it became possible to capture solar energy and use it to produce electricity [3]. In a short period, photovoltaic technology has dominated the market, and now most of the world is shifting to solar energy [4]. However, the major limitation of this technology is its expensiveness and the problems associated with the large-scale production of solar cells as compared to nuclear or fossil fuel energy [5].

**Figure 1:**
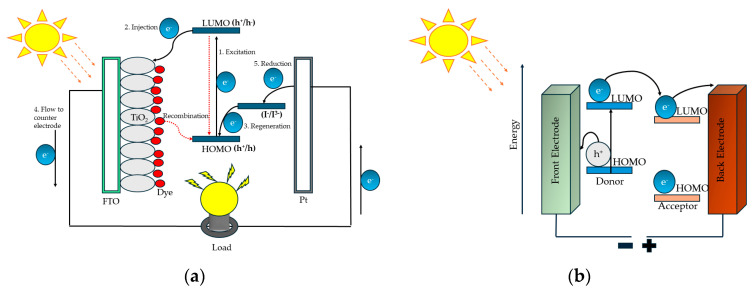
Energy levels and working diagrams of (**a**) dye-sensitized solar cells and (**b**) bulk heterojunction solar cells.

To circumvent the problem associated with the manufacturing of silicon-based solar cells and their high costs, third-generation photovoltaic cells incorporating organic small molecules and polymers were introduced long ago [6,7]. Among them, dye-sensitized solar cells (DSSCs) and organic solar cells (OSCs), also known as bulk heterojunction solar cells (BHJs), have received tremendous attention due to their relatively cheap raw materials, easy fabrication, low cost and light weight [8]. A common feature of these two types of solar cells is that they both incorporate a layer of photoactive material which captures sunlight and converts it into flowing electrons. The photon flux present in solar light shows a maximum around 690 nm (1.8 eV); hence, to absorb light of such wavelengths, materials that have a broad absorption range extending from the visible to near-infrared (IR) regions are required [9,10]. These materials either act as donor (D) or acceptor (A) units blended with a material of opposite nature, or contain donor–acceptor parts in their molecules connected with a π-bridge. In either case, their optical and electrical properties can be fine-tuned by adjusting the frontier orbitals of the donor and acceptor units. The band gaps of such materials are modified as per requirements by the hybridization of the highest occupied molecular orbital (HOMO) of the donor unit with the lowest occupied molecular orbital (LUMO) of the acceptor unit, resulting in widening of the absorption spectra accordingly (cf. Figure 1).

Polymers bearing π-conjugated electrons and certain functional groups attached to the main chain have proved their potential in both DSSCs and BHJ solar cells [11]. When processed properly, the conducting polymers combine their facile processing benefits and excellent mechanical properties with the required optical, electrical, magnetic and electronic properties of metals and metalloids [12,13,14]. The high molar extinction coefficient values and solution processing of such polymers have attracted the interest of researchers seeking to exploit them in these types of solar cells. Their excellent structural stability and tunable optoelectronic properties rationalize their use in solar cells. Such polymers can play various roles according to their structures and resulting properties. In dye-sensitized solar cells, for instance, these polymers can act as a photosensitizer, an electrolyte matrix or even as a counter electrode, while in bulk heterojunction solar cells, they are the fundamental part of the photoactive layer, where they can act as donor or acceptor parts [15,16]. For the successful application of polymers as light absorbers in either type of solar cell, they should bear certain characteristics properties, i.e., (i) they should be sufficiently soluble and processable in common organic solvents, (ii) they must have wide and intense absorption spectra ranging from the visible to near-IR windows, (iii) the energy levels of HOMO and LUMO orbitals should be adjusted to achieve the maximum open-circuit voltage (V_OC_) and hence the overall efficiency of the solar cells, and (iv) they should have high electrical conductivity and (v) an optimum morphology for efficient charge transport and separation [17]. Therefore, structural modifications of polymers should be made to acquire such properties.

**Figure 2:**
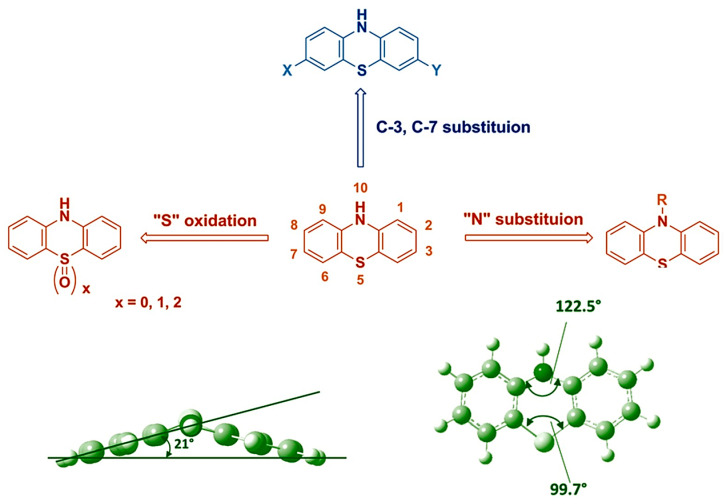
Two- and three-dimensional chemical structures of phenothiazine and its possible functionalization routes [27].

It has been observed that compounds bearing nitrogen atoms, either polymerized or added in small molecular forms as dopants to other polymers, influence the crystalline, optoelectronic and photovoltaic properties of the resulting polymers [18,19,20]. This idea is supported, for instance, by studies where different nitrogenous heterocycles were added to the electrolytes in DSSCs and this resulted in an improvement in the open-circuit voltage (V_OC_), fill factor (FF) and power conversion efficiency (PCE) [21,22]. Similarly, such nitrogen-containing polymers, when exploited as photosensitizers in DSSCs instead of electrolytes, also improved the PV parameters of solar cells [23,24]. In the case of nitrogenous polymers used in bulk heterojunction solar cells, the presence of nitrogen has been observed to broaden the absorption spectra of these polymers as compared to those without nitrogen atoms [25]. Considering these studies, phenothiazine (PTZ) derivatives have been added to polymers either in their main polymeric structures or as dopants. Phenothiazine in particular has attracted the attention of researchers because of its extraordinary optoelectronic properties, which are due to (i) the presence of an extra electron that donates a sulfur atom in addition to nitrogen; (ii) its high conjugation capacity; and (iii) its non-planar butterfly-shaped three-dimensional structure, which prevents aggregation phenomena (cf. Figure 2) [26].

These characteristics of this organic compound make it an ideal candidate for synthesizing intramolecular charge-transfer (ICT) polymers for various optoelectronic devices [28]. Despite the fact that there have been numerous studies in which small phenothiazine derivatives have shown their potential in dye-sensitized solar cells and bulk heterojunction solar cells [29,30], their polymerization can be beneficial too, especially for the thermal and chemical stability of materials and for solar cell devices obtained from these materials. Moreover, the inherent non-planarity of phenothiazine moieties helps in preventing the formation of intermolecular excimers and π-stacking aggregation, thus producing polymers with higher quantum efficiencies [31]. Considering such benefits, polymers that contain phenothiazine as a part of the main chain or as a dopant have been employed in dye-sensitized solar cells and bulk heterojunction solar cells. While carrying out a literature analysis of such studies, it was noted that phenothiazine-based polymers have outperformed many of the other expensive polymers used in DSSCs and BHJ solar cells. Therefore, there is a need to highlight these studies and bring forward the outstanding performance of these polymers so that researchers can work more in this field to further improve the efficiency of DSSCs and BHJ solar cells. To the best of our knowledge, there is no work which has critically reviewed the performance of phenothiazine-based polymers in DSSCs and BHJs in one paper. Therefore, this paper will critically review almost all the studies which have been undertaken so far on the application of phenothiazine-based polymers in dye-sensitized solar cells and bulk heterojunction solar cells. The purpose of this review is to summarize, in one paper, the role of the structures of phenothiazine-based polymers in their optoelectronic, electrochemical and thermal properties. Moreover, the effects of their structures on the photovoltaic performance of DSSCs and BHJ solar cells will be demonstrated. In addition, the effect of the concentration of phenothiazine when used as a dopant in polymer electrolytes will also be reviewed. Polymers containing phenothiazine compounds either as part of the main chain or as dopants in other polymer matrices will be referred to as phenothiazine-based polymers in this work.

## 2. Phenothiazine Polymers in Dye-Sensitized Solar Cells

**Figure 3:**
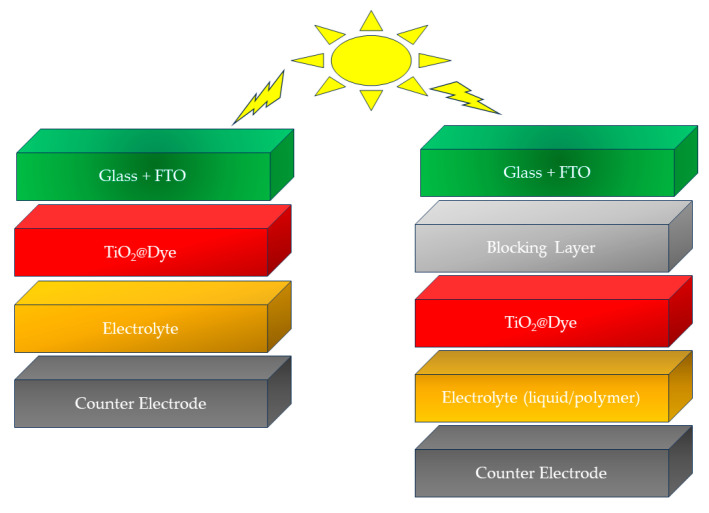
The structure of DSSCs with and without a blocking layer.

Grätzel cells, referred to as dye-sensitized solar cells, are a particular kind of solar cells that use a simple structure and mechanism to convert sunlight into electricity (cf. Figure 3). Dye-sensitized solar cells mimic the photosynthesis phenomena occurring in plants and capture solar photons to convert them into chemical energy [32]. Generally, DSSCs are made up of the following parts with particular chemical compositions:

- Substrate: Metal oxide—usually titanium dioxide (TiO_2_)—nanoparticles are typically employed as semiconductor materials in fabricating photoanodes for DSSCs. Metal oxide nanoparticles in the form of a paste are deposited on a transparent conductive oxide (TCO) surface, such as an indium tin oxide (ITO) or a fluorine-doped tin oxide (FTO) conductive substrate, which is usually made of glass [33,34]. To prevent charge recombination phenomena, a compact layer, also called a blocking layer, is applied before depositing a titanium dioxide layer, which helps to increase the current produced by the dye-sensitized solar cell (cf. Figure 3).
- Photosensitizer: Conjugated donor–acceptor organic or organometallic dye molecules capable of absorbing solar light and producing excited electrons are adsorbed onto the metal oxide surface to prepare the photoanode. Ruthenium-based complexes and organic dyes without metals have all been employed as dyes for DSSCs [35].
- Electrolyte: An electrolyte is injected between the photoanode and the counter electrode, which helps to regenerate the oxidized dye by transferring the electrons to the HOMO of the dye and thus helps in regulating the whole conversion process of the cell. The electrolyte frequently contains iodide/triiodide ions (I^−^/I_3_^−^) or other redox species and can be in liquid, gel or solid form.
- Counter Electrode: The counter electrode is typically made of a conductive glass substrate on which a metal, mostly platinum or a semiconducting polymer like poly(3,4-ethylenedioxythiophene) (PEDOT), is deposited as a catalyst. The catalytic ability of the counter electrode helps in the reduction of electrolyte species. Besides PEDOT, other semiconducting polymers like polypyrrole (PPy), poly(3-hexylthiophene) (P3HT) and polyaniline (PANI) have also been used as counter electrodes in DSSCs.

To capture photons from sunlight, DSSCs employ a light-absorbing dye, frequently a conjugated organic compound which contains an anchoring unit (an acceptor part), which can anchor on the surface of titanium dioxide (cf. Figure 3). Solar light can easily pass through the conductive substrate, which is usually made of a transparent material like glass coated with fluorine-doped tin oxide, to reach the dye layer [36]. The absorption of photons by the dye is accompanied by the simultaneous excitation of its valence electrons from the HOMO of the dye to its LUMO, thus producing an electron–hole pair. The excited electrons from the LUMO of the dye are then injected into the conduction band of titanium dioxide (TiO_2_) via the acceptor part of the dye, i.e., the anchoring unit. Following this, the electrons travel through the TiO_2_ semiconductor towards the conductive substrate, generating an electric current. The use of a blocking layer helps the back-transfer of these electrons from the FTO to the electrolyte ions. Meanwhile, the redox species of the electrolyte are transported to the oxidized dye, where they transfer the extra electrons to the oxidized dye molecules to regenerate a neutral form of the dye, allowing the process to proceed repeatedly. Dye sensitizers are broadly classified into two types: organometallic dyes and metal-free organic dyes. So far, the well-known, commercial ruthenium metal-based organometallic dyes N3, N719 and N749 have proved to be the best-performing DSSC dyes in terms of efficiency and stability. However, metal-free organic dyes have received a lot of interest because of their ease of modification, high molar extinction coefficients, adjustable molecular structures and ecologically friendly composition. DSSCs based on organometallic and purely metal-free organic sensitizers have recently exhibited strikingly comparable sunlight-to-current conversion efficiencies of around 12.5% and 14.3%, respectively [37,38].

**Figure 4:**
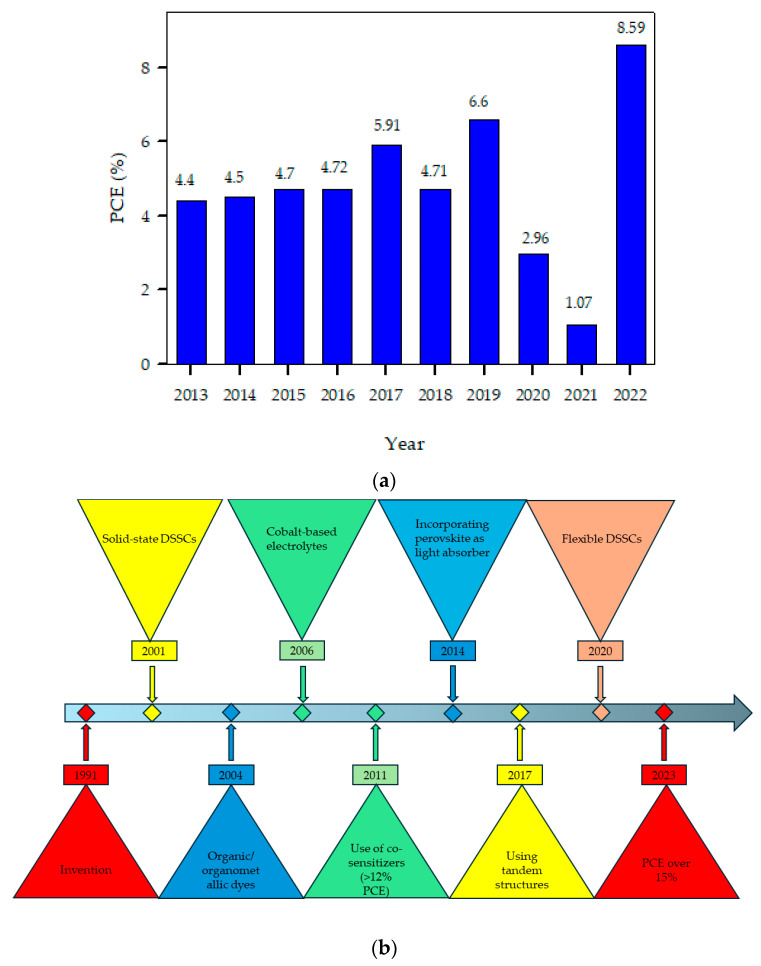
(**a**) Power conversion efficiency (%) of DSSCs employing polymeric photosensitizers over the past ten years and (**b**) the development of DSSCs over time.

Besides small molecules, the use of polymers in DSSCs is also appreciated because they exhibit wide absorption bands compared to small molecules, and their energy levels can be tailored significantly to provide desired electrochemical and photophysical properties which are obligatory for the enhancement of light capture and charge development. Also, the three-dimensional network topologies of polymers rationalize their use as templates to create mesoporous materials or as polymeric matrices in gel electrolytes [39]. Additionally, due to their high catalytic activity for iodide/triiodide ion (I^−^/I_3_^−^) reduction, the polymers may additionally serve as counter-electrode materials [40]. The presence of various functional groups in polymers determines their ability to be applied as interface layers to passivate faults, redefine the functioning of metallic electrodes and enhance device performance [41]. Their high charge carrier mobilities enable polymers to act as both hole- and electron-transport materials [42,43]. Simple solution-based methods, such as spin coating or drop coating, can be used to deposit polymers on conducting substrates to prepare counter electrodes for DSSCs. A technique like spin coating for film deposition is an ideal technique to control the thickness and quality of a film to obtain high power conversion efficiencies. Resultantly, the manufacturing procedure for these polymer-based DSSCs is more straightforward and affordable than it is for conventional silicon solar cells [44]. In short, organic polymers have acquired a remarkable place in DSSC manufacture because they combine the electrical properties of metals with the processing advantages and mechanical properties of polymers. Figure 4a shows the progress made regarding the power conversion efficiency of DSSCs employing polymers as light harvesters over the past ten years. During the literature analysis, it was observed that not many studies have incorporated polymeric dyes into DSSCs, and, from the available data, the most efficient of all polymeric dyes are those which contain phenothiazine in their molecular structures [45,46,47,48,49,50,51,52,53,54,55]. Figure 4b shows the major developmental steps during the entire journey of DSSCs since their invention.

As per our literature review, phenothiazine-based polymers have been exploited in dye-sensitized solar cells as photosensitizers and in electrolyte systems, where phenothiazine has been doped in various polymer matrices to obtain an efficient electrolyte for DSSCs.

### 2.1. Phenothiazine-Based Polymers Applied in Electrolytes in Dye-Sensitized Solar Cells

**Figure 5:**
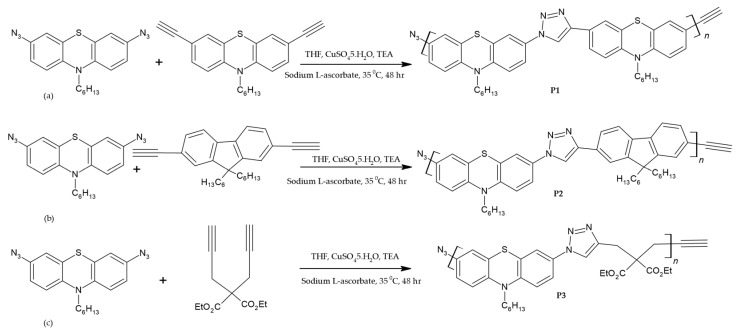
Synthesis scheme for (**a**) P1, (**b**) P2, and (**c**) P3 phenothiazine polymers [61].

Out of the major components of a typical dye-sensitized solar cell, the electrolyte is regarded as the heart of this device because it is responsible for the smooth running of the whole process. Commonly used liquid-based electrolytes containing redox mediators like iodide/triiodide ions have appeared to be the best so far [56]. However, issues related to the leaking and evaporation of liquid electrolytes during the fabrication of DSSCs and the corrosive nature of iodine and its sublimation have compelled scientists to look for better substitutes. To address these issues related to liquid electrolytes, various polymer-based semi-solids, also known as gel electrolytes and solid-state electrolytes, have been developed [57]. However, the relatively poor conductivity of solid-state electrolytes limits their application, and a lot of work is needed to improve the PV parameters of devices employing this type of electrolytes. Alternative approaches, such as the use of polymer gel electrolytes made of single polymers, copolymers and polymer blends, seem to be more efficient. To further enhance the conductivity and decrease the crystallinity of such polymers, doping of organic compounds like propylene carbonate, ethylene carbonate, diethyl carbonate and organic nitrogenous compounds and the addition of various other nanofillers have been employed [22,58,59,60]. Polymer-based electrolytes are some of the best choices because of their good interfacial filling properties, excellent ionic conductivity and long-term stability. One of the major advantages of polymer electrolytes is that they are easy to synthesize from readily available and cheap monomers and can give higher charge-transport abilities. Considering these features, Song, M., et al. [61] synthesized phenothiazine-based click polymers (P1, P2 and P3) using Cu(I)-catalyzed click reactions and exploited them as polymer matrices for electrolytes in DSSCs (cf. Figure 5). To ensure the purity of these polymers for their better performance, the precipitating click polymers were refined further using repeated Soxhlet extractions with methanol, followed by chloroform extraction.

The resultant click polymers exhibited solution processability because of their good solubility [61]. All these polymers had good thermal stability and thus could eliminate the problem of electrolyte degradation during cell functioning. They had absorption maxima around 350 nm, which implies that their absorption range will not interfere with the absorption window of photosensitizers and therefore that they can be used as matrices to prepare electrolytes. While preparing polymer electrolytes for DSSCs, iodine (I_2_) and tetrabutylammonium iodide (TBAI) were chosen to generate redox ion pairs. For comparison, polyacrylonitrile (PAN) (M_W_ = 86,200) was also used as a matrix, and the photovoltaic performances of DSSCs using a SnO_2_:F/TiO_2_/N719 dye/polymer electrolyte/Pt device architecture were compared. Under typical solar illumination conditions (one sun), the maximum power conversion efficiency achieved using the P3 electrolyte was 5.30%, which was even higher than the DSSC employing a polyacrylonitrile-based electrolyte (cf. Table 1). The higher photocurrents produced by the phenothiazine polymers were due to their low molecular weights as compared to the PAN. Even though this type of synthesis of polymers is facile, it still took a lot of time and various reagents to obtain polymers of high purity. Therefore, an alternative way to improve the performance of DSSCs using polymer electrolytes is to add phenothiazine as a dopant in the polymer matrix. This strategy is based on the fact that nitrogen-containing heterocycles are known to increase the V_OC_ of DSSCs by interacting with I^−^/I_3_^−^ redox couples [62]. Moreover, the addition of non-planar dopants also causes the crystallinity of polymers to decrease, which can be beneficial to increase the conductivity of these polymers.

Following this claim, Amudha, S., et al. [63] synthesized a polymer electrolyte by adding phenothiazine in a blend of polymethylmethacrylate (PMMA) and polyvinylidene fluoride (PVDF), while potassium iodide (KI) and iodine (I_2_) were used to generate redox couples (I^−^/I_3_^−^). The authors studied the effect of the concentration of phenothiazine present in this electrolyte system on the PV parameters of DSSCs, which varied as follows: 0, 0.004, 0.009, 0.014, 0.019, 0.024, 0.029 and 0.034 g. XRD studies revealed that the addition of PTZ resulted in a decrease in the crystallinity of this copolymer, which caused an upsurge in the ionic mobility of the resultant electrolyte. This increase in the ionic conductivity was also evidenced by the improved electrical conductivity after the addition of phenothiazine—4.5 × 10^−7^ Scm^−1^ for the undoped polymer blend and 4.5 × 10^−6^ Scm^−1^ for the blend containing 0.004 g PTZ. The reason for such an increase in the conductivity was attributed to the increase in the free volume of the electrolyte after the doping of phenothiazine [64]. It is probably for this reason that the DSSC incorporating the electrolyte with the 0.004 g PTZ component showed the highest PCE of 4.8% as compared to the undoped electrolyte, the PCE of which was around 1.4% (cf. Table 1). The PV parameters shown in Table 1 clearly indicate that the addition of phenothiazine in the polymer matrix increased both the V_OC_ and J_SC_ values of the DSSCs. This is attributed to the fact that the lone pair present on the nitrogen of phenothiazine formed a charge-transfer complex with the redox pair in the electrolyte and prevented the sublimation of iodine, thereby increasing the photocurrent density and the voltage produced by the cell.

**Table 1:** Photovoltaic parameters of DSSCs employing phenothiazine-based polymer electrolytes.

Composition of Polymer	V_OC_ (mV)	J_SC_ (mAcm^−2^)	FF (-)	PCE (%)	Ref.
^a^ P1/TBAI/I_2_/PMII/EC:PC(3:1)	600	12.84	0.56	4.38	[61]
P2/TBAI/I_2_/PMII/EC:PC(3:1)	600	14.56	0.55	4.84	
P3/TBAI/I_2_/PMII/EC:PC(3:1)	600	14.25	0.62	5.30	
PAN/TBAI/I_2_/PMII/EC:PC(3:1)	600	12.32	0.55	4.05	
PMMA/PVDF/KI/I_2_	550	2.50	0.48	1.40	[63]
PMMA/PVDF/KI/I_2_/0.004 g PTZ	820	5.80	0.50	4.80	
PMMA/PVDF/KI/I_2_/0.009 g PTZ	740	3.30	0.48	2.30	
PMMA/PVDF/KI/I_2_/0.014 g PTZ	720	3.00	0.48	2.10	
PMMA/PVDF/KI/I_2_/0.019 g PTZ	740	3.50	0.44	2.30	
PMMA/PVDF/KI/I_2_/0.024 g PTZ	750	3.60	0.42	2.20	
PMMA/PVDF/KI/I_2_/0.029 g PTZ	650	2.80	0.49	1.80	
PMMA/PVDF/KI/I_2_/0.034 g PTZ	760	3.90	0.40	2.40	
PVDF-PEO/KI/I_2_	650	7.50	0.50	3.50	[65]
PVDF-PEO/KI/I_2_ DPA	790	10.20	0.52	6.00	
PVDF-PEO/KI/I_2_ PTZ	850	13.20	0.53	8.50	
PVDF/KI/I_2_	587 ± 5.8	3.43 ± 0.20	0.42	1.42	[66]
PTZ-PVDF/KI/I_2_	616 ± 4.7	5.00 ± 0.17	0.57	2.92	

^a^: TBAI = tetrabutylammoniumiodide, PMII = 1-propyl-3-methylimidazolium iodide, EC = ethylene carbonate, PC = propylene carbonate.

Ganesan, S., et al. [65] also followed the same direction and prepared two novel polymer electrolytes by doping phenothiazine and diphenylamine (DPA) separately in a PVDF-poly(ethylene oxide) (PEO) blend. The reason for replacing PMMA with PEO was that PEO had a better miscibility with PVDF as compared to PMMA. The polymer electrolyte containing phenothiazine as the filler had the lowest degree of crystallinity, which ultimately translated into the highest electrical conductivity of 3.3 × 10^−4^ Scm^−1^ for this electrolyte as compared to the undoped and DPA-containing electrolytes, whose values were 6.9 × 10^−5^ and 1.6 × 10^−4^ Scm^−1^, respectively. After their complete characterization, these copolymers were then used to prepare two different polymer electrolyte systems, i.e., PVDF/PEO/KI/I_2_/DPA and PVDF/PEO/KI/I_2_/PTZ, while one electrolyte, i.e., PVDF/PEO/KI/I_2_, was prepared without any dopant for reference purposes. The observance of an absorption maximum around 394 nm indicated the better interaction of phenothiazine with iodine in the PVDF/PEO/KI/I_2_/PTZ electrolyte as compared to the DPA-based electrolyte system. For the fabrication of DSSCs, the Cis-dithiocyanato-bis (2,2 bipyridyl 4,4 dicarboxylic acid) ruthenium (II) complex (N3 dye) was used, and three DSSC solar cells in the (a) TiO_2_/N3dye/KI/I_2_/Pt, (b) TiO_2_/N3dye/KI/I_2_/DPA/Pt and (c) TiO_2_/N3dye/KI/I_2_/PTZ/Pt device architectures were prepared. It was observed that the V_OC_ was 850 mV in the case of the PTZ-containing electrolyte, while it was 790 mV for the DPA-containing electrolyte. However, in both cases, the open-circuit voltage was higher than that of the electrolyte without any plasticizer (cf. Table 1). Generally, it is observed that nitrogenous compounds interact with the iodide/triiodide redox couple through the lone pair present at the nitrogen atom; however, in the case of phenothiazine, an additional electron-donor sulfur atom also contributed to the better interaction with the redox couple as compared to DPA. The sulfur atom of phenothiazine assisted in the formation of a charge-transfer complex with iodine, which led to an increase in the voltage and current produced by the DSSC using the phenothiazine electrolyte. The overall PCEs of the three DSSCs under 70 m Wcm^−2^ were 3.50, 6.00 and 8.50%, respectively, for cells utilizing undoped, DPA-doped, and phenothiazine-doped polymer electrolytes.

Besides using a polymer blend, a single conducting polymer can be a potential matrix for polymer electrolytes when doped with a nitrogenous compound. Senthil, RA., et al. [66] studied phenothiazine as a dopant in PVDF/KI/I_2_ electrolytes, for which they used a solution casting process to prepare PVDF/KI/I_2_ electrolyte films doped with PTZ in various weight percentage (wt. %) ratios (0, 20, 30, 40 and 50%), using DMF as a solvent. According to the XRD analysis, the value of crystallinity (X_C_) for the undoped electrolyte was the highest among all the samples, i.e., 63.80, while the electrolyte with the 20% phenothiazine content had the lowest. The value of crystallinity went on increasing with the increase in the phenothiazine percentage, which in turn was attributed to the rise in the number of uncoordinated species in the polymer matrix. The lowering of the X_C_ value translated into an increase in ionic mobility, and thus the electrolyte with the 20% phenothiazine content had the lowest X_C_ value and hence showed the highest electrical conductivity. AC-impedance analysis showed that the undoped PVDF/KI/I_2_ electrolyte had an ionic conductivity of 4.68106 Scm^−1^, which increased dramatically upon PTZ addition to 7.43105 Scm^−1^. The 20% PTZ-PVDF/KI/I_2_ electrolyte had the strongest ionic conductivity, outperforming the other wt. % compositions. Therefore, this polymer electrolyte was chosen for DSSC fabrication. Using the optimized weight percentage of the PTZ-doped PVDF/KI/I_2_ electrolyte in a DSSC, a significant power conversion efficiency of 2.92% was attained, compared to 1.41% for the undoped PVDF/KI/I_2_ counterpart. As a result, under identical conditions, the 20% PTZ-PVDF/KI/I_2_ electrolyte emerged as a suitable contender for DSSC applications. Compared with the undoped electrolyte, the DSSC employing the electrolyte with dopped phenothiazine showed higher photovoltaic parameters. The higher mobility of ions of redox species (I^−^/I_3_^−^) in this electrolyte resulted in higher J_SC_ and V_OC_ values, leading to an increase in the overall efficiency of the solar cell (cf. Table 1).

It is evident from the above studies that polymer electrolytes doped with phenothiazine or containing phenothiazine as part of their structure can be potential substitutes for the currently used liquid electrolytes. The excellent thermal properties, higher conductivities and non-propensity to electrolyte leakage are properties which make these polymers ideal for use as electrolytes in DSSCs. Further work should be carried out to improve the conductivities of polymer matrices to compete with the performance provided by liquid electrolytes.

### 2.2. Phenothiazine-Based Polymers as Photosensitizers in Dye-Sensitized Solar Cells

A photosensitizer, also called a dye, is the main component of dye-sensitized solar cells because it captures the sunlight and, as a result, produces free electrons which then travel across the whole circuit. Phenothiazine-based polymers have also been utilized as photosensitizers in DSSCs due to their higher molar extinction coefficient values and high charge-transport capabilities. The effective intramolecular charge transfer from the donor part of a photosensitizer to its acceptor part is critical to obtain high PV parameters. To increase ICT phenomena, two types of strategies can be adopted in the case of phenothiazine-based polymers. Either the phenothiazine can be incorporated into the backbone of the polymer, which leads to an increase in the π-conjugation, or a polymeric antenna can be attached to the phenothiazine, where the phenothiazine acts as a side-chain donor. The latter approach is also useful to obtain polymeric photosensitizers with excellent optoelectronic and electrochemical properties because the side group, e.g., three-dimensional phenothiazine, affects the packing of polymeric chains, and thus the physical properties of the polymers are greatly influenced [67]. Depending on the position of phenothiazine in the structure of these polymers, they can be divided into two types for better understanding, i.e., (a) phenothiazine in the main chain and (b) phenothiazine in the side chain. However, no obvious difference was observed in the PV parameters of DSSCs employing any of these types.

#### 2.2.1. Phenothiazine in the Main Chain

**Figure 6:**
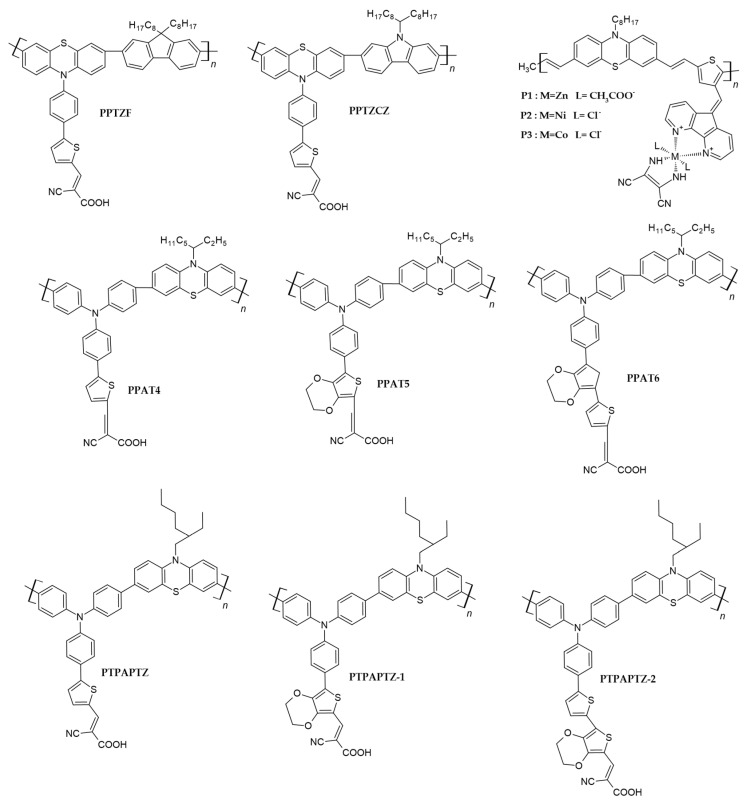
The chemical structures of phenothiazine polymers employing PTZ in the main chain for DSSCs.

Inspired by the performance of small phenothiazine dyes, Tan, H., et al. [45] synthesized two phenothiazine-based polymer dyes, PPTZF and PPTZCZ, containing cyanoacrylic acid as an anchoring unit (cf. Figure 6). Another triphenylamine-based polymer, PTPACZ, was also synthesized by replacing phenothiazine with the triphenylamine unit in PPTZCZ for comparison purposes. PPTZF and PPTZCZ showed absorption around 330 and 370 nm; however, the molar extinction coefficient of PPTZCZ, which contained 9-(heptadecan-9-yl)- 9H-carbazole in its backbone, was almost double that of PPTZF, which contained 9,9-dioctyl-9H-fluorene in its structure. To obtain photoanodes, a 7 + 5 µm thick titanium dioxide bilayer was used to deposit the dye in a THF–acetonitrile (1:1) solution mixture. Under 100 mWcm^−2^, the overall efficiency of PPTZF, which contained a DSSC, was 3.0%, while in the case of PPTZCZ, the PCE was 3.5% (cf. Table 2). This increase in the PCE of the latter dye was due to the high open-circuit voltage (V_OC_) and current density (J_SC_) values shown by this dye, which were attributed to its comparatively high dielectric constant values. It is generally observed that the degree of polymerization of these polymers has a direct influence on the photovoltaic parameters of dye-sensitized solar cells. The polymers with the lowest degree of polymerization have the highest incident photon-to-converted electron (IPCE) and overall PCE values [68]. This was also obvious in this study, where PPTZF, with a molecular weight of 3.249 kgmol^−1^, had higher PV parameters as compared to PPTZCZ, which had a molecular weight of 3.021 kgmol^−1^.

Combining the properties of metals and polymers in “metallopolymers” not only yields materials with outstanding properties, such as high conductivities and excellent mechanical and thermal stabilities, but also reduces the costs associated with using expensive metals exclusively. It is generally observed that electrons can efficiently be transferred from the conjugated polymer main chain to the metal complex, bonded either in the main chain or in the side chain. Unlike blending two polymers, this approach of combining a metal with a polymer can also decrease intrinsic defects like phase separation. Considering these advantages, Xie, Q., et al. synthesized branched-chain polymeric metal complexes (P1, P2 and P3) comprising phenothiazine and thiophene derivatives and exploited them as photosensitizers for DSSCs (cf. Figure 6) [69]. In the polymer structure, phenothiazine appended with an octyl chain served as the donor (D) and a C=C bond served as the bridging element, while thiophene–phenanthroline metal complexes were used as an acceptor (A), allowing anchoring of the dye onto the TiO_2_ surface. The presence of a phenothiazine atom made the electron-donating part stronger, and its non-planar structure inhibited the formation of excimers. The phenothiazine-based metallopolymers combined the thermal properties of metals and their electrochemical characteristics, i.e., HOMO–LUMO levels were in accordance with the requirements for DSSCs. Under AM 1.5G solar irradiation simulation, the sensitizer P1 showed the highest energy conversion efficiency of 1.57% (Jsc = 4.12 mA/cm^2^, Voc = 620 mV, FF = 0.61) (cf. Table 2). It can be noted from the photovoltaic parameters of these polymers that the J_SC_ values were constantly low. The reason for such low parameter values is the poor anchoring of these polymers to the titanium dioxide surface as compared to the carboxyl group. Furthermore, the open-circuit voltages were found to be 620, 570 and 540 mV for the P1, P2 and P3 polymers, which changed in accordance with the HOMO values of the polymers. It was therefore concluded that the structure of phenothiazine and the attached anchoring unit has a major impact on the overall efficiency of DSSCs. These findings highlight the critical importance of molecular engineering and establish the groundwork for the development of novel conjugated organic polymer dyes, which will aid in the development of highly efficient and long-lasting DSSCs [69].

**Figure 7:**
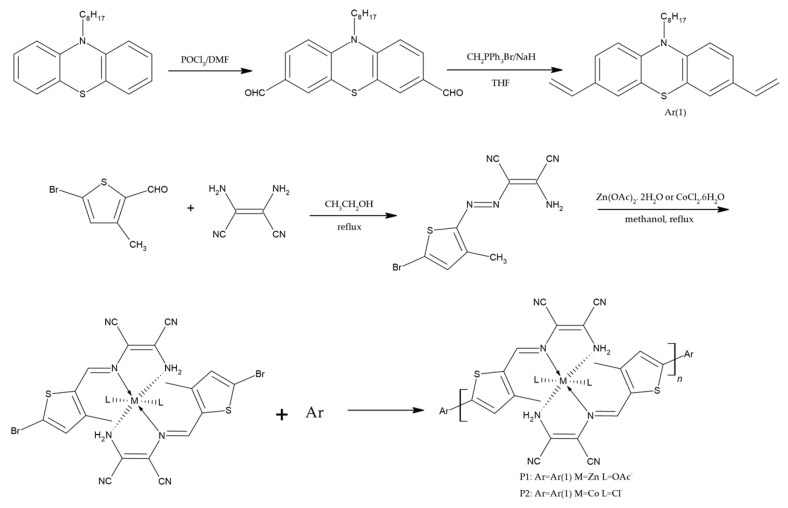
Synthesis of polymeric complexes of P1–P2 [70].

This study by Xie, Q., et al. [69] showed that the introduction of transition metal ions into conducting polymers is a key approach for obtaining materials with unique optical and electrochemical properties. Therefore, following in their footsteps, Chen, X., et al. [70] also developed and studied stable main-chain polymeric metal complex dyes with phenothiazine units (P1 and P2), with an emphasis on their potential for dye-sensitized solar cells (cf. Figure 7). These dyes demonstrated outstanding chemical stability and resistance to high temperatures, as TGA and DSC studies showed the higher thermal stability of these dyes, with T_g_ values of 162 and 147 °C for P1 and P2, respectively. The dyes containing PTZ units had wider absorption spectra as compared to the metal complexes without PTZ donors. Notably, the DSSC system sensitized with P1 demonstrated the highest photo-current conversion efficiency, reaching 1.88% under typical lighting circumstances. A short-circuit photocurrent density of 4.30 mAcm^−2^, an open-circuit photo voltage of 640 mV and a fill factor of 0.68 demonstrated this overall PCE (cf. Table 2).

The addition of π-spacers between donor and acceptor parts improves the light harvesting ability of photosensitizers by extending the π-conjugation in the structures [71]. Inspired by the potential of phenothiazine as a three-dimensional donor and the role of thiophene as a π-spacer described in the literature, Wang, G., et al. [47] synthesized three conjugated poly(triphenylamine-phenothiazine)-based polymers which consisted of a side chain containing a thiophene unit (PPAT4) alternated with either a 3,4-ethylenedioxythiophene (EDOT, PPAT5) or an EDOT-thiophene (PPAT6), which served as the π-bridge, and cyanoacetic acid, which acted as the anchoring unit (cf. Figure 6). A small conjugated compound (PAT) was also synthesized for reference purposes. Absorption spectra showed that the molecular extinction coefficient values of the polymers were very high as compared to PAT, and PPAT6 exhibited the highest value. The electrochemistry of these polymers proved that the LUMOs of these phenothiazine polymers were less negative as compared to the conduction band gap of TiO_2_, which is the among the basic requirements for materials to be used as dyes for DSSCs. Furthermore, the extension of the π-conjugation in the structure led to an increase in the electron-donating ability of these dyes. Under AM 1.5G conditions, the highest PCE of 4.7% was shown by PPAT4, while all of the polymers showed higher conversion efficiencies than the small conjugated compound (cf. Table 2). It can be noted that by polymerizing the small phenothiazine derivative PAT to give PPAT4, the PCE was increased by up to 64% as compared to PAT.

A similar attempt was made by Xiong, S., et al. [50], where three phenothiazine-based conjugated polymers featuring triphenylamine and phenothiazine as donor-conjugated units, thiophene and 3,4-ethylenedioxythiophene as π-spacers and cyanoacrylic acid as side-chain acceptors were synthesized and exploited as sensitizers in dye-sensitized solar cells. Keeping the rest of the structure similar, PTPAPTZ exhibited thiophene as a π-spacer and PTPAPTZ-1 contained 3,4-ethylenedioxythiophene as a π-spacer, while PTPAPTZ-2 contained both thiophene and EDOT as π-spacers (cf. Figure 6). Due to the extension of π-conjugation in the PTPAPTZ-2 polymer featuring two π-spacers, the ICT spectrum of this polymer was very broad and had the maximum molar extinction coefficient. Moreover, this polymer had the narrowest band gap, which resulted in an improved light harvesting ability due to the extended π-conjugation. The power conversion efficiencies of the DSSCs obtained from these phenothiazine-containing polymers were 4.08, 3.66 and 4.71% for PTPAPTZ, PTPAPTZ-1 and PTPAPTZ-2, respectively (cf. Table 2). PTPAPTZ-1 had the lowest PCE because it had higher LUMO levels and the widest band gap, which resulted in a decrease in the electron injection from the HOMO of this polymer to the conduction band of TiO_2_, thereby decreasing the photocurrent density (J_SC_).

#### 2.2.2. Phenothiazine in the Side Chain

**Figure 8:**
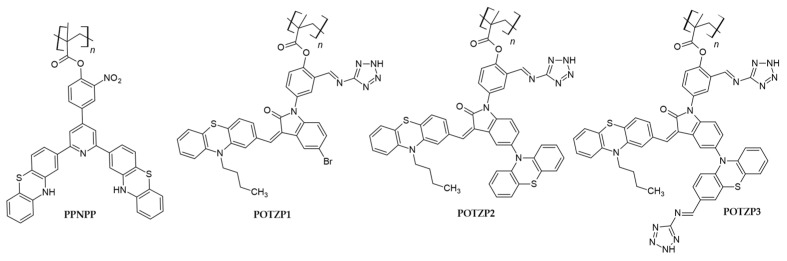
The chemical structures of phenothiazine polymers employing PTZ in the side chain for DSSCs.

Ramasamy, S., et al. [49] designed three π-spacer-containing methacrylate polymers, POTZP1, POTZP2 and POTZP3, featuring phenothiazine derivatives bearing a butyl chain at the N10 position as a donor, oxindole as a π-spacer and a tetrazole ring as an acceptor (cf. Figure 8). The obtained polymers had higher thermal stabilities up to 305 °C. The absorption spectra of these phenothiazine polymers in DMF solution showed two absorption bands: a short-wavelength, higher-energy band, which was attributed to π-π* transition, and a low-energy, longer-wavelength band, which was due to intramolecular charge transfer between the acceptor and donor parts of the polymers. By introducing a phenothiazine moiety between the oxindole and tetrazole anchoring units in the case of POTZP3, the ICT band was redshifted to about 30 nm as compared to the other two polymers.

The band gaps of these polymers were in the following order: POTZP1 > POTZP2 > POTZP3. This drop in the band gap was probably due to the specific assembly of the phenothiazine donors and tetrazole acceptors, which resulted in the widening of the absorption spectra and thus caused an increase in the light harvesting efficiency. While adsorbed on the TiO_2_ surface, these phenothiazine polymers showed a blueshift in their absorption maxima, which was possibly due to the H-aggregation and deprotonation of the tetrazole ring. The LUMO levels of all the polymers were less negative as compared to the band gap of the titanium dioxide (−4.0 eV vs. vacuum), while the HOMO levels were more negative (lower in energy) than the redox energy of the I^-^/I_3_^-^ couple (−4.6 eV vs. vacuum). Such electrochemical and optical studies justify the use of these polymers in the fabrication of DSSCs. Under AM 1.5 solar light (100 mWcm^−2^), the highest efficiency of 5.91% was achieved for POTZP3, as per expectations from optical and electrochemical studies. The presence of two anchoring units in this polymer decreased the HOMO–LUMO gap, and thus electron injection was increased from the HOMO of POTZP3 to the conduction band of titanium dioxide, giving a current density of 11.45 mAcm^−2^, a V_OC_ of 850 mV and a fill factor of 0.74 (cf. Table 3).

Prakash, G., et al. [72] studied three polymers, one of which, PPNPP, exhibited phenothiazine as the electron-donor part and pyridine as the π-spacer, while nitrobenzene served as the electron acceptor/anchoring group (cf. Figure 8). The other two polymers, FPNPP and APNPP, contained fluorine and anthracene in place of phenothiazine in their structures. Differential scanning calorimetry showed that the polymer bearing the phenothiazine donor had the maximum T_g_ value, i.e., 152, and the maximum decomposition value of 302 °C as compared to FPNPP and APNPP. PPNPP showed absorption maxima at 360 nm, which was attributed to π-π* transition, while a low-energy ICT band was observed around 430 nm. After adsorption on a titanium dioxide surface, a blueshift of 8 nm was observed in the ICT spectrum of this polymer, which was due to the H-aggregation and deprotonation of the anchoring group. The oxidation potential of PPNPP as determined from cyclic voltammetry was 690 mV, which was higher than that of the fluorine- and anthracene-containing polymers. The phenothiazine-containing polymer had the most positive oxidation potential due to the strong donating abilities of phenothiazine. Similarly, the electrochemical band gap (E_g_) of this polymer was 2.78 eV, which was the lowest out of the three polymers. Two types of DSSCs, i.e., one with and one without a co-adsorbent (CDCA), were prepared from these polymers. The devices employing PPNPP as a photosensitizer showed power conversion efficiencies of 4.12% and 2.18% with and without the co-adsorbent, respectively, which were higher than those of the devices employing polymers containing fluorine and anthracene donors (cf. Table 3).

The above-mentioned studies concluded that it will be more convenient to develop long-term, efficient DSSC devices based on phenothiazine polymers. The results show that by modifying the structure of these polymers, the optical and electrochemical properties can be tuned as per requirements. The materials required to synthesize phenothiazine polymers are readily available and reasonably priced, which may help make the manufacture of DSSCs more affordable. By utilizing renewable resources and producing clean electricity, dye-sensitized solar cells, particularly those that incorporate ecofriendly materials like phenothiazine-based polymers, have the potential to enhance green energy production [49]. To fully realize the promise of phenothiazine polymers in DSSCs, it is crucial to keep in mind that while they present intriguing possibilities, there may also be some associated difficulties, and therefore there is a need for continued research in this field. The research should be focused on enhancing the long-term stability of DSSCs and on decreasing any potential toxicity or conservational issues associated with the components. To circumvent these issues, phenothiazine-based polymers can be helpful because they have implications for improved stability, adaptability and efficiency enhancements of DSSCs. They can facilitate advancement in the development of solar energy technologies and their potential integration into real-world applications.

## 3. Phenothiazine Polymers in Bulk Heterojunction Solar Cells

**Figure 9:**
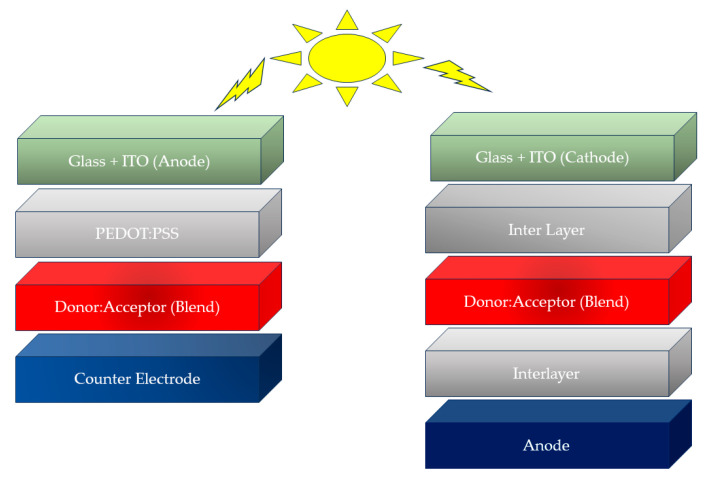
Schematic structures of BHJ solar cells with conventional and inverted structures.

Bulk heterojunction solar cells utilize a bulk of donor and acceptor materials as an active layer, and the architecture of these solar cells is designed to maximize the interfacial area between the donor and acceptor materials, allowing for more effective charge separation and transmission (cf. Figure 9) [26]. The sequential processes of light absorption, charge creation, charge separation and charge collection underpin the operation of BHJ solar cells: photons from the sun are absorbed by the active layer as they strike the solar cell, producing excitons (electron–hole pairs) within the donor and acceptor materials (cf. Figure 1). Because of the wide interfacial areas of donor–acceptor materials and their closeness to each other, the excitons in the bulk heterojunction structure are more prone to dissociation into free charges (electrons and holes) at the interface between the donor and acceptor materials. The separated charges then migrate through the donor and acceptor materials towards their respective electrodes (often a transparent conductive electrode and a metal electrode), providing an electric current that can be collected and used as electrical energy [73].

**Figure 10:**
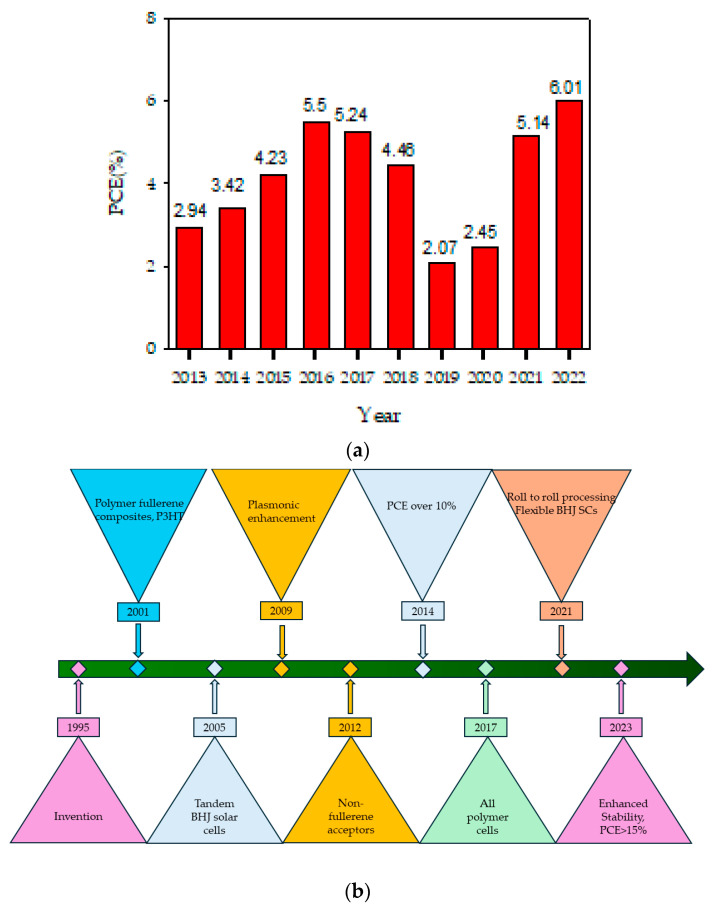
(**a**) Power conversion efficiency (%) of BHJ solar cells containing P3HT:PCBM over the past 10 years and the (**b**) development of BHJ solar cells over time.

The subject of organic solar cells is constantly evolving, with researchers experimenting with different material combinations to improve efficiency and stability [74]. Because of their low band gaps and good charge-transport capabilities, conjugated polymers, among others, have become “state-of-the-art” donor materials in bulk heterojunction (BHJ) solar cells. Currently, the most commonly used active layer for BHJ solar cells consists of a poly(3-hexylthiophene) acceptor and [6,6]-phenyl-C_61_-butyric acid methyl ester as a donor, giving overall power conversion efficiencies of about 5–6% [75]. However, there is still a need to improve the efficiency of BHJ solar cells by using novel donor and acceptor units. Therefore, scientists are continuously attempting to develop efficient donor–acceptor polymers with narrow band gaps to efficiently harvest visible light. One approach to obtain narrow-band gap polymeric materials is to copolymerize the donor and acceptor polymers so that, in the copolymers, the charge can be efficiently transferred from the donor polymer to the acceptor polymer [76]. Another way to realize low band gaps even in homopolymers is the modification of their units either by adding heterocyclic donors (like thiophene, pyrrole and phenothiazine) and acceptors (benzothiadiazole, dioxythiophene and thienopyrazine), or by introducing conjugated side chains, which approach is helpful in avoiding relatively difficult copolymer synthesis [77,78,79,80]. Figure 10 shows the power conversion efficiencies for the last ten years and major developments made in bulk heterojunction solar cells since their invention [81,82,83,84,85,86,87,88,89].

**Figure 11:**
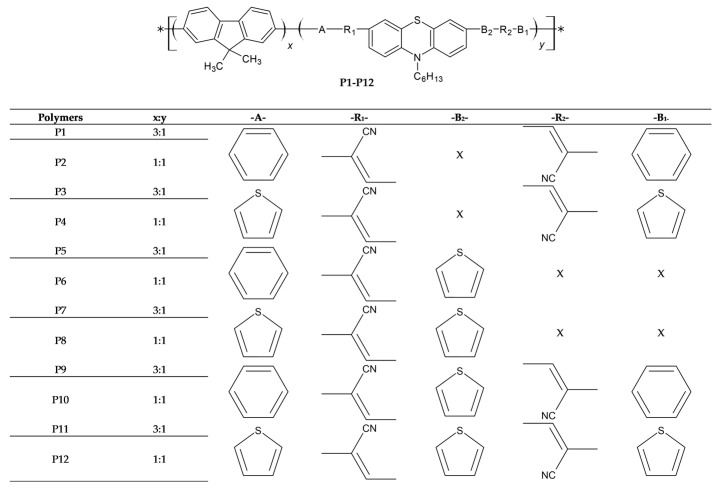
Structure of phenothiazine polymers synthesized by Li, KC. et al. [90].

Impressed by the versatile electrochemical and optical properties of phenothiazine, Li, KC., et al. [90] synthesized twelve phenothiazine-containing narrow-band gap copolymers (FO-PT) by incorporating 9,9-dihexylfluorene- (FO) and phenothiazine-based heteroarylene–cyanovinylene derivatives (PTs), where two series of copolymers were synthesized by varying the ratio of the two parts in the FO-PT copolymers (cf. Figure 11). Gel permeation chromatography (GPC) analysis showed that the number-average molecular weight of the polymers was in the range of 8400 to 27,900, while the average molecular weights ranged from 10,800 to 102,900. One common absorption band around 375 nm present in all the copolymers except P4 and P12 came from poly(9,9-dihexylfluorene) units, while the second low-energy, broad-absorption band was due to the presence of extended conjugation caused by phenothiazine units. As the content of PTZ units increased in the copolymers, the ratio of absorbance of PTZ to fluorene units also increased. Moreover, a higher phenothiazine content led to redshift of the long-wave absorption band, which was attributed to the higher conjugation that occurred between the PTZ units as compared to the fluorene units. The copolymers containing a higher ratio of PT, i.e., FO_1_-PT (1:1), exhibited a broad absorption range of 200–800 nm and narrow band gaps as compared to those copolymers which contained less phenothiazine, i.e., FO_3_-PT (3:1). The LUMO levels of these copolymers were in the range of 3.28–3.54 eV, and if they were blended with PCBM acceptors, they had the ability to transfer charge to the PCBM, which had a LUMO of 4.2 eV.

Owing to the broader absorption window and the narrow band gap of FO_1_-PT as compared to FO_3_-PT, the former series of copolymers were chosen as donors to fabricate bulk heterojunction solar cells in devices with ITO/PEDOT:PSS/**FO_1_-PT:PCBM**/LiF/Al architectures. A thin film of the active material showed complete PL quenching for the P12 copolymer after it was blended with PCBM as P12:PCBM. The maximum results were shown by the P12:PCBM (1:4) mixture, which had a power conversion efficiency of 0.51% (cf. Table 4). The lower photovoltaic parameters of these copolymers were possibly due to the low thickness and disordered morphology of the film. FO_1_-PT polymers with low molecular weights resulted in the films having a lower thickness, and thus the number of photons harvested was also very low. Therefore, the short-circuit current density was only between 1.30 and 2.70 mAcm^−2^. Generally, V_OC_ values are associated with the difference between the oxidation potential of the donor and the reduction potential of the acceptor; all the copolymers followed this rule, except P12. These lower photovoltaic parameters can be improved by modifying the morphology of the thin film, optimizing the thickness of the active layer or using different acceptors.

**Figure 12:**
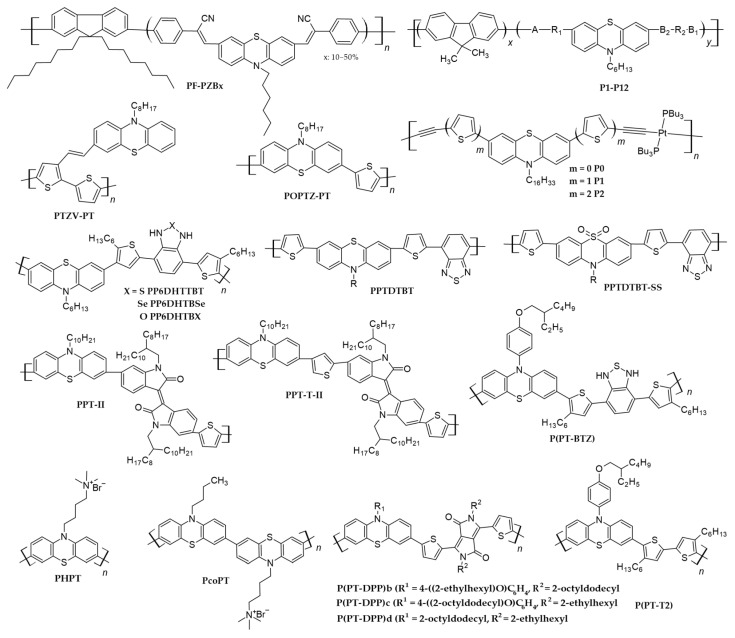
Structures of phenothiazine polymers for bulk heterojunction solar cells.

If phenothiazine polymers are to be included in BHJ solar cells, it is better to use them as blended electron-donor materials because phenothiazine has electron-donating capabilities and therefore has the potential to improve the absorption and charge-transport properties of organic solar cells. The journey of phenothiazine-based polymers as donors in BHJ solar cells began accidently when Cho, NS., et al. [91] synthesized copolymers incorporating phenothiazine in their polymer structures to improve their electroluminescence and hole-transporting properties. The actual aim of this study was to obtain red-light-emitting phenothiazine polymers, and, for this purpose, the authors synthesized five copolymers, viz., PF-PZBx, by varying the percentage of bis(2-phenyl-2-cyanovinyl)-10-hexylphenothiazine) (PZB) units in the resulting copolymers from x = 10 to 50% (cf. Figure 12). The copolymers thus obtained exhibited number-average molecular weights (M_n_) in the range of 13,000 to 24,000, and the presence of phenothiazine units in the backbones of these copolymers increased their absorption by between 430 and 630 nm, which was attributed to the increase in conjugation caused by the phenothiazine units. The band gap (2.14 eV) of this polymer compelled the researchers to try it as a donor in bulk heterojunction solar cells. They blended PF-PZB50 polymers with [6,6]-phenyl C61 butyric acid methyl esters (PCBM), and photoluminescence spectra showed complete emission quenching as compared to pristine PF-PZB50. Considering emission quenching phenomena, bulk heterojunction solar cells were fabricated by exploiting the thin film obtained from the solution of this blend in chlorobenzene, using spin coating, in the ITO/PEDOT-PSS/**PF−PZB50:PCBM(1:3)**/LiF/Al architecture, which gave a power conversion efficiency of 0.53% under an AM 1.5 solar simulator with V_OC_ and J_SC_ values approaching 780 mV and 2.38 mAcm^−2^, respectively. This study pioneered the use of phenothiazine polymers as donors in BHJ solar cells and suggested that the photovoltaic parameters of these solar cells can be further improved by varying the donor–acceptor ratios and utilizing acceptors other than PCBM.

The adjustment of the band gaps of polymers to be used in BHJ solar cells is a topic of immense importance, as the open-circuit voltage (V_OC_) of these solar cells is primarily determined from their band gaps. It has been experimentally determined that the band gap value for these polymers should be between 1.2 and 1.92 eV, corresponding to HOMO energy levels between −5.8 and −5.2 eV and LUMO levels between −4.0 and −3.8 eV. Keeping these facts in view, Padhy. H., et al. synthesized three novel donor–acceptor polymers containing phenothiazine as the donor and benzodiazole as the acceptor parts [92]. Various polymers, viz., PP6DHTBT, PP6DHTBSe and PP6DHTBX, which contained benzothiadiazole, benzoselenodiazole and benzoxadiazole, respectively, sandwiched between hexylthiophene linkers (cf. Figure 12), were investigated as donors in bulk heterojunction solar cells. The GPC analysis showed that the number-average molecular weights of the polymers were 4.07 × 10^4^, 5.13 × 10^4^ and 3.85 × 10^4^, while the weight-average molecular weights were 7.54 × 10^4^, 10.17 × 10^4^ and 6.45 × 10^4^, respectively, for PP6DHTBT, PP6DHTBSe and PP6DHTBX. The polymer containing benzoxadiazole showed the maximum absorption intensity, while the derivative containing a selenium atom had the maximally redshifted spectrum, which was probably due to the bigger size of the selenium and its electron-rich nature. The band gap values calculated using electrochemistry and photophysical studies were 1.93, 1.80 and 1.90 for PP6DHTBT, PP6DHTBSe and PP6DHTBX, respectively. When these phenothiazine polymers were blended with PCBM, they showed emission quenching phenomena, indicating their feasibility for BHJ solar cells. BHJ solar devices bearing ITO/PEDOT:PSS (30 nm)/polymer:PCBM blend (∼80 nm)/Ca (30 nm)/Al (100 nm) structures were prepared. When the blend was prepared with PC_61_BM, the maximum PCE was about 0.41% for the cell containing PP6DHTBT as the donor material. Based on the results, this polymer was chosen and blended with PC_71_BM in different weight ratios, showing PCEs of 0.74% for the 1:1 ratio and 1.20% for the 1:4 ratio. The increase in PCBM weight led to improvement in the photovoltaic parameters of the solar cells, which was due to the increase in the roughness caused by the higher PCBM content, as was shown by AFM studies. This study also concluded that optimizing the amounts of donors and acceptors in the blend, changing the acceptors, and improving the roughness of the active layer can improve the efficiency of BHJ solar cells.

Following this research, Kim, G., et al. [28] synthesized two polymers, viz., PPTDTBT and PPTDTBT-SS, by copolymerizing electron-deficient benzothiadiazole with electron-donating phenothiazine and phenothiazine-S,S-dioxide, producing copolymers with number-average molecular weights of 9.8 × 10^3^ and 7.6 × 10^3^ g/mol, respectively (cf. Figure 12). The former polymer showed a relatively low hole mobility of 9.8 × 10^–5^ cm^2^V^–1^s^–1^, while the polymer containing the oxidized form of phenothiazine showed a hole mobility of 6.9 × 10^–4^ cm^2^V^–1^s^–1^. Optical band gaps of 1.79 eV and 1.95 eV were obtained for the PPTDTBT and PPTDTBT-SS polymers, respectively. The reason for the higher optical band gap can be recognized by considering the electron-withdrawing sulfonyl groups in the structure of the latter polymer, which decrease its electron-donating ability. Two types of conventional and inverted BHJ solar cells with ITO/PEDOT:PSS/polymers:PC_71_BM/Al structures were fabricated by blending these donor polymers with PC_71_BM in the weight ratios of 1:1 and 1:4. The optimum weight ratio for PPTDTBT was 1:2, while for PPTDTBT-SS it was 1:1.5 (cf. Table 4 and Table 5). The short-circuit current density of PPTDTBT in conventional and inverted devices was 5.75 and 4.80 mAcm^−2^, respectively. Regarding the inverted structure, a decrease in the short-circuit current density was observed for PPTDTBT, a possible reason for which was the absorption of a fraction of light by the top Au electrode. However, in the case of PPTDTBT-SS, the J_SC_ was improved for the inverted architecture. A possible reason for this was the presence of sulfonyl groups in the polymer which facilitated the contact between the two electrodes. Atomic force microscopy images also support the results obtained after measuring the morphology of the active layer. The results showed that the PPTDTBT:PCBM blend was very uniform, while the PPTDTBT-SS:PCBM blend was not very homogenous. This work also concluded that increasing the solubility of the polymers in common organic solvents assists in the formation of more homogenous active layers and thus can improve the overall PCE of solar cells.

In another study [93] aiming to improve the PCE of bulk heterojunction solar cells, a successful attempt was made by the authors, who synthesized two copolymers based on phenothiazine and isoindigo (cf. Figure 12). The presence of a thiophene linker in PPT-T-II resulted in an increase in the co-planarity between the donor and acceptor parts; therefore, the intramolecular charge-transfer spectrum of PPT-T-II was shifted to a longer λ_max_. PPT-II was soluble in common organic solvents, while PPT-T-II was not soluble in any solvent except hot chloroform; therefore, only the number-average molecular weight of the soluble portion could be determined, which was 7900 g/mol, while for PPT-II, this value was 55,000 g/mol. The low solubility of the former polymer was probably due to the rigid backbone or due to the presence of thiophene units. However, due to the insolubility of this polymer, BHJ solar cells were fabricated only from the PPT-II polymer in the device with the ITO/PEDOT:PSS/PPT-II:PC_71_BM/LiF/Al configuration. The ratios of donor to acceptor blends varied from 1:1 to 1:4, which resulted in an improvement in the device efficiency from 0.09% to 0.50% (cf. Table 4). The increase in the J_SC_ value with the increase in PCBM in the blend contributed mainly to the increase in PCE. The reason for the improvement in the current density by increasing the PCBM ratio was due to the increase in the light absorption and the better PPT-II/PCBM interface. To further improve the photovoltaic parameters, the active layer containing the 1:4 donor–acceptor blend was thermally annealed to 150 °C. The AFM images showed that the morphology of the thermally annealed blend was improved, and thus it resulted in an improvement of PCE from 0.5 to 0.74%, again due to an increase in the short-circuit current density. Hence, blending phenothiazine-based polymers with PCBM and thermally treating the active layer can help to obtain cost-effective, high-efficiency BHJ devices.

In 2022, Wessling, R., et al. [98] investigated the multifarious properties of conjugated copolymers based on phenothiazine as electron-donor and -acceptor units. Their research included testing these copolymers as positive-electrode materials for lithium–organic batteries, as well as determining their performance in bulk heterojunction (BHJ) photovoltaic devices. Benzothiadiazole (BTZ) and diketopyrrolopyrrole (DPP) acceptors were used in the production of D-A-type copolymers, yielding P(PT-BTZ) and P(PT-DPP)b polymers, respectively, while a phenothiazine–bithiophene copolymer, P(PT-T2), which had a purely donor nature, was also tested for comparison. Two further derivatives of the P(PT-DPP)b polymer, i.e., P(PT-DPP)c and P(PT-DPP)d, were also synthesized by varying alkyl chain lengths (cf. Figure 12). The ITO/ZnO/BHJ/MoOx/Ag structure was adopted for BHJ solar cells, where ZnO and MoO_x_ were used as electron-transport and hole-transport layers, respectively. Both fullerene acceptors, PC_60_BM and PC_70_BM, and non-fullerene acceptors, O-IDFBR and ITIC, were used to study the effect of the nature of acceptors on the overall efficiency of BHJ cells. The PCE shown by blending P(PT-T2):ITIC was 0.08%, while for P(PT-DPP)b:O-IDFBR, the PCE was 0.05%. However, for the fullerene acceptors, P(PT-DPP)d/PC_71_BM (1:3), the efficiency increased by up to 1.87% (cf. Table 5).

Copolymerization of two homopolymers is not only the way to obtain narrow-band gap materials; such polymers can also be obtained by introducing side chains along the backbones of homopolymers. To actualize this concept, Sang, G., et al. [94], synthesized two polymers, one of which, viz., POPTZ-PT, had a phenothiazine moiety copolymerized with polythiophene, while the other had phenothiazine units connected to the polythiophene chain as side groups (PTZV-PT) (cf. Figure 12). The PTZV-PT polymer had a weight-average molecular weight of 5.4 × 10^4^, while for POPTZ-PT, this value was 3.4 × 10^4^, and the polydispersity indexes were 1.48 and 1.72, respectively. The thermal decomposition temperature of these polymers was around 400 °C, which shows their excellent thermal properties. The absorption spectrum of the polymer (PTZV-PT) containing phenothiazine units as side substituents on the polythiophene backbone was broader and covered a wider wavelength than the polymer (POPTZ-PV) which incorporated PTZ units in its backbone. The PTZV-PT polymer had a narrow band gap of 1.81 eV, while the band gap of POPTZ-PV was around 2.26 eV; hence, the approach that lowered the band gap of the polymers by side substitution of phenothiazine was more effective than the copolymerization approach. The space-charge-limited current (SCLC) method indicated a higher hole mobility of 4.7 × 10^−3^ cm^2^V^−1^s^−1^ for PTZV-PT as compared to 5.0 × 10^−4^ cm^2^V^−1^s^−1^ for POPTZ-PV. The broad absorption spectrum and higher hole mobility of the former polymer were expressed in its better performance in BHJ solar cells as compared to the latter. The polymer bulk heterojunction solar cells fabricated with the device architecture as ITO/PEDOT:PSS/polymer:PCBM/Au, gave PCEs of 1.0% for PTZV-PT and 0.09% for POPTZ-PV (cf. Table 4). Due to similar HOMO values, both polymers delivered similar open-circuit voltages upon photoexcitation. The low fill factor (FF) of the solar cells was probably due to the morphology of the thin film and the interface between the electrode and the active layer. The lower J_SC_ of POPTZ-PT was due to its narrow absorption window as compared to PTZV-PT. It was concluded that, by device optimization, the PCE of the cell exploiting PTZV-PT can be improved.

Nevertheless, PCBM is so far the most widely adapted polymer acceptor in BHJ solar cells. The low absorption characteristics of this polymer limit its efficiency, and therefore it is desirable to synthesize an alternative substitute for this polymer. Considering this problem, in their next studies [95], the authors synthesized a poly[1,4-dioctyloxyl-p-2,5-dicyanophenylenevinylene] (DOCN-PPV) acceptor and used it with PTZV-PT from previous studies to prepare all-polymer BHJ solar cells. The absorption spectrum of the polymeric blend was a superposition of the individual absorption spectra of PTZV-PT and PCBM. Photoinduced charge transfer in the blend was observed, as evidenced by the PL quenching during photoluminescent spectroscopy. The device structure opted for was ITO/PEDOT:PSS/PTZV-PT:DOCN-PPV (1:1, w/w)/LiF(~3–5 Å)/Al(~100 nm), which showed a PCE of 0.4%, which further increased to 0.8% after annealing to 120 °C (cf. Table 4). This efficiency value was close to the value obtained when PCBM was used as an acceptor. The study concluded that photovoltaic parameters can be further improved by modifying the morphology of the active layer or changing the acceptor.

Instead of all-organic polymers, scientists have also tried using metal-containing conjugated polymers for energy conversion and obtained excellent results [99,100]. To obtain narrow-band gap metallopolymers, Wong, WY., et al. [96] incorporated platinum and phenothiazine units to make alternating copolymers (cf. Figure 12). The effect of thiophene units was studied in three synthesized polymers (P0, P1 and P2) with varying thiophene units in the phenothiazine cores. GPC analysis using polystyrene standards showed that their number-average molecular weights were in the order of 11,350 to 16,070. The absorption spectra of these polymers showed absorption maxima in the range of 349–430 nm, and from these photophysical studies the band gaps of P0, P1 and P2 were calculated to be 2.90, 2.66 and 2.52 eV, respectively. Upon blending with PCBM acceptors, all three polymers showed emission quenching phenomena. Considering these characteristics, two types of BHJ solar cells were fabricated using these polymers in the architectures of ITO/PEDOT:PSS/polymer:PCBM (1:4) and (1:5)/Al, in which the donor-to-acceptor ratios were changed. It can be seen from Table 4 that the short-circuit current density of the P2 polymer was higher than that of the P1 polymer, which was also expected from the wide absorption spectrum of the latter polymer. These results showed that an increase of 0.2–0.3% can be achieved by increasing the number of thienyl units along the backbone, which results in an increase in the absorption coefficient. Both hole and electron mobilities can also be increased by increasing “m” in the polymer structure (cf. Figure 12). The maximum PV performance of 1.29% was shown by the polymer bearing two thiophene units in the PTZ core and blended with PCBM in the ratio of 1:5. It is further expected that optimizing the donor–acceptor blend composition and device construction will lead to the betterment of device stability and efficiency.

Besides their use as donors, phenothiazine polymers have also been tested as conducting polymer electrolytes (CPEs) in bulk heterojunction solar cells by Jo, MY., et al. [97]. They synthesized two phenothiazine polymers, viz., PHPT and PcoPT, the former containing quaternary ammonium salts on each side chain of the phenothiazine ring, while the latter bore salt ions on the side chains of the phenothiazine ring (cf. Figure 12). They investigated the effect of the side-chain arrangements and the electronic properties on the PV parameters of BHJ solar cells. Ultraviolet photoelectron spectroscopy (UPS) showed that the work functions of the PHPT- and PcoPT-coated aluminum electrodes were 3.92 eV and 3.73 eV, respectively, which were lower than the work function of the bare Al electrode (4.16eV). ITO/PEDOT/P3HT:PCBM/CPE/Al was adopted to fabricate BHJ devices from PHPT and PcoPT, while one reference cell was also fabricated for comparison with the ITO/PEDOT/P3HT:PCBM/Al structure. The power conversion efficiencies of PHPT and PcoPT were 2.69 and 2.96, respectively, which values were higher than that obtained for the reference device with a bare Al electrode, i.e., 2.47 (cf. Table 4). An increase in the open-circuit voltage (V_OC_) played a major role in this improvement in PCE, which in turn was due to a decrease in the work function of the electrode in the case of the CPE.

The research work in this field reviewed above shows that phenothiazine polymers may have a broad scope as donors in bulk heterojunction solar cells because of their wide absorption spectra, which allow them to catch a greater range of solar wavelengths, depending on their specific chemical structures. This results in better light harvesting and, possibly, higher power conversion efficiencies for BHJ solar cells [101]. Furthermore, studies have proved that polymers based on phenothiazine are solution processable, allowing them to be deposited on conducting substrates via facile techniques such as spin coating, inkjet printing and roll-to-roll coating. Because BHJ solar cells are compatible with solution-based manufacturing processes, they can be mass-produced on a large scale and with a low budget [102]. When utilized as polymer components, phenothiazine polymers have demonstrated strong thermal and photochemical stability, which may add to the durability of BHJ solar cells [103]. The experimental work reported in the above-cited studies showed that phenothiazine polymers are relatively simple to synthesize and modify, allowing researchers to tune their characteristics to specific device requirements. This synthetic adaptability has the potential to lead to the development of innovative materials with improved performance characteristics [27]. It should be noted that the consequences of utilizing phenothiazine polymers in BHJ solar cells are dependent on a variety of factors, including the specific polymer structure, complementary material selection, device architecture and overall processing conditions. Organic photovoltaics researchers continue to investigate and optimize the usage of phenothiazine-based polymers and other new materials to improve the efficiency, stability and commercial viability of organic solar cells [27].

## 4. Summary and Conclusions: Challenges, Solutions and Future Prospects

Phenothiazine polymers have shown considerable promise in dye-sensitized solar cells and organic solar cells, but as with any technology, they are not without problems. There are certain challenges associated with the manipulation of these polymers in DSSCs and BHJ solar cells which need to be addressed and solved to obtain high PCEs for these devices using phenothiazine-based polymers. The challenges include the difficulty of aligning the energy levels of phenothiazine polymers with those of other components in the solar cell stack. Mismatches in energy levels can result in poor charge transfer and losses. Moreover, phenothiazine polymers, like many organic materials, can be susceptible to environmental conditions, which can alter their stability and performance over time. Compared to other conducting polymers, phenothiazine polymers may have comparatively limited solar spectrum absorption, which may result in poor light harvesting and reduced overall efficiency. Hence, chemical-structure optimization of these polymers is required to widen their absorption range [92,104]. Furthermore, it is critical for large-scale manufacturing to develop cost-effective and scalable synthesis methods for phenothiazine polymers, as well as appropriate processing procedures.

**Figure 13:**
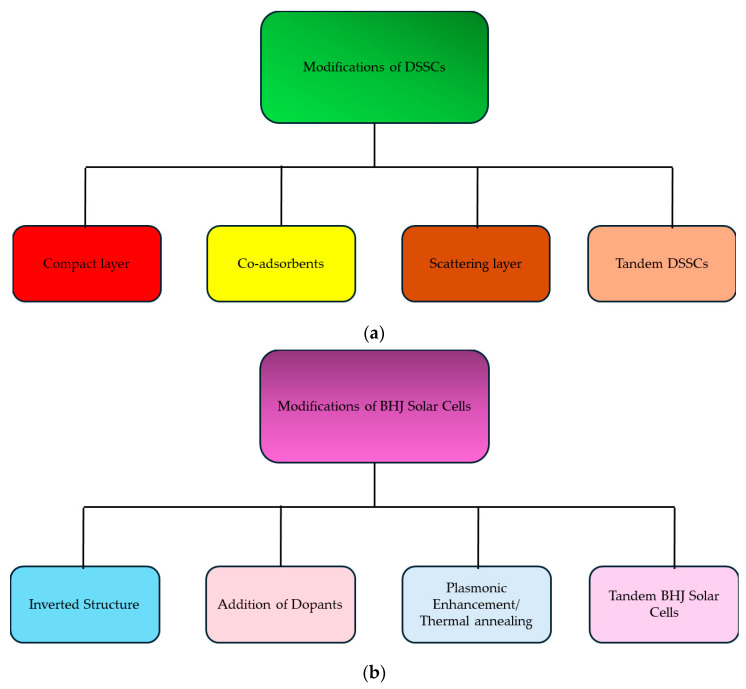
Possible modifications to improve the PCE of (**a**) DSSCs and (**b**) BHJ solar cells using phenothiazine polymers.

Solutions to these common problems include fine-tuning the energy levels of phenothiazine polymers by altering their chemical structures, ensuring better alignment with other materials and lowering energy losses. Figure 13 shows the possible modifications which can be made in DSSCs and BHJ solar cells to further improve PV parameters using phenothiazine-based polymers.

Continued research into more efficient and cost-effective synthesis methods for phenothiazine polymers will most certainly result in enhanced materials that are easier to produce on larger scales. Strategies such as encapsulation, interface engineering and the introduction of stabilizing chemicals might assist improvements to the stability of phenothiazine-based devices and hence extend their operating lifetimes. Researchers are looking into ways to broaden the absorption range of phenothiazine polymers by designing materials that can absorb light in a larger spectrum and hence enhance overall light harvesting [90]. Phenothiazine polymers can be used in tandem or multi-junction solar cell topologies, which combine various materials with complementary absorption characteristics to improve the total efficiency of these PV devices. When phenothiazine polymers are combined with other sophisticated materials, such as new electron-transporting materials and perovskite absorbers, device performance can be improved synergistically. Furthermore, computational approaches can assist in forecasting the properties of novel phenothiazine polymers, allowing researchers to build materials with desired qualities more efficiently. The unusual electrical and optoelectronic properties of these polymers suggest a bright future for their use in solar cells.

In short, phenothiazine-based polymers have a strong potential to substitute the currently used metals and other expensive polymers. The development of every technology takes many years; therefore, if research is focused on solving the issues related to low device efficiency while manipulating these polymers, the time is not far away when the world will experience facilities of low-cost energy production. Now, ongoing research focuses on increasing the stability, scalability and synthesis methods of phenothiazine-based polymers to make them viable candidates for next-generation organic solar cells. Phenothiazine polymers may contribute to improving the efficiency and cost-effectiveness of solar energy conversion. Additionally, with the advancement of material engineering and device architectures, these polymers can help in the long-term expansion of renewable energy technology.

This article has given an introduction to and in-depth assessment of current breakthroughs in the use of conducting phenothiazine-based polymers in DSSCs and BHJ solar cells, covering the benefits of phenothiazine polymers for improving solar cell performance, as well as the problems encountered during their practical integration and potential solutions to these challenges. Notably, because of their broad set of qualities, such polymeric materials can be deliberately used to design many efficient optoelectronic devices. Photosensitizers and electrolytes for dye-sensitized solar cells can be obtained using phenothiazine polymers which have intrinsic abilities to outperform other polymers in terms of efficiency. These polymers can also be useful as interface layers, hole-transport materials and electron-transport materials, enhancing charge carrier separation and inhibiting recombination phenomena. Phenothiazine-based polymers serve critical roles in organic solar cells as donor layers, acceptor layers and electrolytes with the goal of affecting device outputs. As the field of material engineering and device design evolves, phenothiazine polymers will figure as contributors in efforts to increase the efficiency and cost-effectiveness of solar energy conversion. Therefore, they will play an important role in promoting the long-term growth and advancement of renewable energy technology.

## Figures and Tables

**Table 2:** Photovoltaic parameters of DSSCs employing phenothiazine-based polymer photosensitizers with PTZ in the main chain.

Polymer	V_OC_ (mV)	J_SC_ (mAcm^−2^)	FF (-)	PCE (%)	Ref.
^a^ PPTZF	742	5.30	0.77	3.0	[45]
PPTZCZ	775	6.06	0.75	3.5	
PTPACZ	769	8.12	0.71	4.4	
^b^ P1	620	4.12	0.61	1.57	[69]
P2	570	3.64	0.63	1.31	
P3	540	3.77	0.60	1.22	
^b^ P1	640	4.30	0.68	1.88	[70]
P2	610	4.21	0.66	1.70	
^a^ PAT	660	6.91	0.66	3.0	[47]
PPAT4	720	10.87	0.60	4.7	
PPAT5	700	8.05	0.65	3.7	
PPAT6	650	9.80	0.64	4.1	
^a^ PTPAPTZ	650	9.80	0.75	4.08	[50]
PTPAPTZ-1	700	8.05	0.82	3.66	
PTPAPTZ-2	720	10.87	0.78	4.71	

Solvents for photoanode preparation: ^a^: THF–acetonitrile (1:1) and ^b^: DMF.

**Table 3:** Photovoltaic parameters of DSSCs employing phenothiazine-based polymer photosensitizers with PTZ in the side chain.

Polymer	V_OC_ [mV]	J_SC_ [mAcm^−2^]	FF	PCE [%]	Ref.
^a^ POTZP1	740 ± 0.03	5.90 ± 0.04	0.68 ± 0.02	3.97 ± 0.03	[49]
POTZP2	780 ± 0.01	8.83 ± 0.02	0.61 ± 0.00	4.42 ± 0.01	
POTZP3	850 ± 0.01	11.45 ±0.01	0.74 ± 0.02	5.91 ± 0.01	
^b^ PPNPP	710	4.35	0.68	2.18	[72]
* PPNPP	760	7.23	0.69	4.12	

Solvent for photoanode preparation: ^a^: DMF, ^b^: tertiary butylalcohol:acetonitrile (1:1) * with CDCA.

**Table 4:** Photovoltaic parameters of conventional BHJ solar cells incorporating phenothiazine-based polymers.

Device Architecture	V_OC_ (mV)	J_SC_ (mAcm^−2^)	FF (-)	PCE (%)	Ref.
ITO/PEDOT-PSS/**PF−PZB50**(1):PCBM(3)/LiF/Al	780	2.38	0.29	0.53	[91]
ITO/PEDOT:PSS/**P2**(1):PCBM(4)/LiF/Al	430	1.86	0.27	0.22	[90]
ITO/PEDOT:PSS/**P6**(1):PCBM(4)/LiF/Al	520	1.46	0.22	0.17	
ITO/PEDOT:PSS/**P8**(1):PCBM(4)/LiF/Al	270	2.21	0.27	0.16	
ITO/PEDOT:PSS/**P10**(1):PCBM(4)/LiF/Al	530	1.30	0.26	0.18	
ITO/PEDOT:PSS/**P12**(1):PCBM(1)/LiF/Al	550	2.10	0.25	0.29	
ITO/PEDOT:PSS/**P12**(1)PCBM(2)/LiF/Al	560	2.30	0.28	0.36	
ITO/PEDOT:PSS/**P12**(1):PCBM(4)/LiF/Al	640	2.70	0.29	0.51	
ITO/PEDOT:PSS/**PP6DHTBT**(1):PC_61_BM(1)/Ca/Al	670	1.92	0.32	0.41	[92]
ITO/PEDOT:PSS/**PP6DHTBSe**(1):PC_61_BM(1)/Ca/Al	650	1.43	0.30	0.28	
ITO/PEDOT:PSS/**PP6DHTBX**(1):PC_61_BM(1)/Ca/Al	690	1.24	0.29	0.25	
ITO/PEDOT:PSS/**PP6DHTBT**(1):PC_71_BM(1)/Ca/Al	730	2.95	0.34	0.74	
ITO/PEDOT:PSS/**PP6DHTBT**(1):PC_71_BM(3)/Ca/Al	730	3.80	0.33	0.88	
ITO/PEDOT:PSS/**PP6DHTBT**(1):PC_71_BM(4)/Ca/Al	750	4.60	0.35	1.20	
ITO/PEDOT:PSS/**PPTDTBT**(1):PC_71_BM(2)/Al	770	5.75	0.38	1.69	[28]
ITO/PEDOT:PSS/**PPTDTBT-SS**(1):PC_71_BM(1.5)/Al	810	4.03	0.30	0.97	
ITO/PEDOT:PSS/**PPT-II**(1):PC71BM(1)/LiF/Al	790	0.47	0.24	0.09	[93]
ITO/PEDOT:PSS/**PPT-II**(1):PC71BM(2)/LiF/Al	650	1.9	0.26	0.33	
ITO/PEDOT:PSS/**PPT-II**(1):PC71BM(3)/LiF/Al	650	2.4	0.26	0.40	
ITO/PEDOT:PSS/**PPT-II**(1):PC71BM(4)/LiF/Al	640	2.5	0.27	0.50	
^a^ ITO/PEDOT:PSS/**PPT-II**(1):PC71BM(4)/LiF/Al	660	3.3	0.26	0.58	
^b^ ITO/PEDOT:PSS/**PPT-II**(1):PC71BM(4)/LiF/Al	680	3.6	0.26	0.64	
^c^ ITO/PEDOT:PSS/**PPT-II**(1):PC71BM(4)/LiF/Al	700	3.9	0.28	0.74	
ITO/PEDOT:PSS/**POPTZ-PT**(1):PCBM(1)/Au	700	0.38	0.32	0.09	[94]
ITO/PEDOT:PSS/**PTZV-PT**(1):PCBM(1)/Au	730	4.03	0.34	1.0	
ITO/PEDOT:PSS/**PTZV-PT**(1):DOCN-PPV(1)/LiF/Al	850	3.14	0.28	0.80	[95]
ITO/PEDOT:PSS/**P1**(1):PCBM(4)/Al	630	2.73	0.32	0.55	[96]
ITO/PEDOT:PSS/**P1**(1):PCBM(5)/Al	780	3.71	0.37	1.06	
ITO/PEDOT:PSS/**P2**(1):PCBM(4)/Al	730	3.95	0.30	0.86	
ITO/PEDOT:PSS/**P2**(1):PCBM(5)/Al	790	4.06	0.41	1.29	
ITO/PEDOT/P3HT:PCBM/**PHPT**/Al	610	7.41	0.59	2.69	[97]
ITO/PEDOT/P3HT:PCBM/**PcoPT**/Al	610	8.02	0.60	2.96	

^a^: annealing temperature: 80 °C; ^b^: annealing temperature: 100 °C; ^c^: annealing temperature: 120 °C.

**Table 5:** Photovoltaic parameters of inverted BHJ solar cells incorporating phenothiazine-based polymers.

Device Architecture	V_OC_ (mV)	J_SC_ (mAcm^−2^)	FF (-)	PCE (%)	Ref.
ITO/PEDOT:PSS/**PPTDTBT**(1):PC_71_BM(2)/Al	780	4.80	0.39	1.47	[28]
ITO/PEDOT:PSS/**PPTDTBT-SS**(1):PC_71_BM(1.5)/Al	920	4.11	0.32	1.22	
ITO/ZnO/**P(PT-T2)**(1)/PC61BM(1.5)/MoOx/Ag	788	0.54	0.32	0.14	[98]
ITO/ZnO/**P(PT-DPP)b**(1)/PC61BM(1.5)MoOx/Ag	677	1.73	0.35	0.40	
ITO/ZnO/**P(PT-DPP)c**(1)/PC61BM(1)MoOx/Ag	497	4.57	0.39	0.89	
ITO/ZnO/P**(PT-DPP)d**(1)/PC61BM(3)MoOx/Ag	757	6.12	0.40	1.87	
ITO/ZnO/P**(PT-BTZ)**(1)/PC61BM(1)MoOx/Ag	675	1.80	0.29	0.36	

## Data Availability

Not applicable.
